# Cognitive Behavioural Therapy for Insomnia in Psychiatric Disorders

**DOI:** 10.1007/s40675-016-0055-y

**Published:** 2016-10-20

**Authors:** Markus Jansson-Fröjmark, Annika Norell-Clarke

**Affiliations:** 1 Department of Psychology, Stockholm University, SE-106 91 Stockholm, Sweden; 2 Centre for Research on Child and Adolescent Mental Health, Karlstad University, SE-651 88 Karlstad, Sweden

**Keywords:** Insomnia, CBT, Mood disorders, Anxiety, PTSD, Psychosis

## Abstract

Insomnia means difficulties in initiating or maintaining sleep and is commonly comorbid with psychiatric disorders. From being considered secondary to primary psychiatric disorders, comorbid insomnia is now considered an independent health issue that warrants treatment in its own right. Cognitive behavioural therapy for insomnia (CBT-I) is an evidence-based treatment for insomnia. The effects from CBT-I on comorbid psychiatric conditions have received increasing interest as insomnia comorbid with psychiatric disorders has been associated with more severe psychiatric symptomologies, and there are studies that indicate effects from CBT-I on both insomnia and psychiatric symptomology. During recent years, the literature on CBT-I for comorbid psychiatric groups has expanded and has advanced methodologically. This article reviews recent studies on the effects from CBT-I on sleep, daytime symptoms and function and psychiatric comorbidities for people with anxiety, depression, bipolar disorder, psychotic disorders and post-traumatic stress disorder. Future strategies for research are suggested.

## Introduction

Insomnia is a common health complaint that means difficulties in falling asleep, maintaining sleep, or waking up too early in the morning [1]. The symptoms of insomnia disorder also include daytime impairment, such as tiredness, and/or distress. Insomnia is frequently comorbid with psychiatric diagnoses [2, 3] and when it is comorbid, it has been associated with more severe psychiatric symptomology [4–6] and higher odds of new psychiatric episodes (e.g. [7, 8]). Previously, insomnia was viewed as secondary to a primary psychiatric diagnosis, with the causal assumption that the sleep problems would remit with successful treatment of the primary disorder. Nowadays, comorbid insomnia is viewed as an independent health problem that warrants treatment in its own right, which is also reflected in how diagnostic criteria no longer distinguish between primary or secondary insomnias [1, 9].

Cognitive behavioural therapy for insomnia (CBT-I) is an evidence-based treatment of insomnia that can be delivered in several formats: as individual therapy, group therapy, internet therapy or self-help (e.g. [10, 11]). Independent of treatment format, the therapies are usually short and they rarely exceed eight sessions or 2 months. CBT-I includes a combination of behavioural and cognitive techniques in order to change maladaptive sleep habits, to lower sleep-disrupting arousal (cognitive or physiological) and to alter sleep-related misconceptions and thought patterns. Studies have demonstrated that, for primary insomnia, CBT-I is equal to pharmacological treatment in the short-term and has longer lasting effects [12, 13]. Further, a recent meta-analysis indicates that the effects of CBT-I on insomnia comorbid with various medical or psychiatric disorders are similar as for primary insomnia [14]. A related promising finding is that CBT-I has been associated with improvements in comorbid symptomologies, such as depressive severity, thus numerous reviews have focused on the possible extended effects on psychiatric symptoms [15–20].

While we acknowledge the contributions made by previous researchers, a new review is warranted to provide an update based on studies that have been published in recent years; many of which take on new types of patient groups and use novel and/or stronger research designs than previously published. There is great variation in how much research has been conducted on the effects from CBT-I on different psychiatric diagnoses, and caution is warranted when generalising to under-investigated patient groups. Thus, our aim was to provide an updated summary (a) of the effects of CBT-I in psychiatric disorders with a particular focus on the following outcomes: insomnia symptoms, domains specific to psychiatric disorders, daytime symptoms and function and (b) to suggest implications for future research.

## CBT-I in Psychiatric Disorders

### Anxiety Disorders

To date, no trials have been published on the efficacy of CBT-I among those with anxiety disorders and comorbid insomnia. There are, however, some encouraging data that suggest that CBT-I might reduce anxiety symptoms. First, a meta-analysis on the effects of CBT-I on anxiety symptoms showed that the combined effect was small to moderate (Hedges’ *g* = 0.41) [21]. There was a similar effect for those patients that fulfilled criteria for a clinical diagnosis or reported substantial anxiety problems [Hedges’ *g* = 0.47; i.e. post-traumatic stress disorder (PTSD), excessive pre-sleep arousal and high trait anxiety] and those who did not display significant anxiety symptoms (Hedges’ *g* = 0.40). Second, another meta-analysis suggest that internet-delivered CBT-I is moderately (Cohen’s *d* = 0.35) effective in reducing anxiety symptoms, as assessed by validated anxiety scales [22•]. A notable caveat with the second meta-analysis is, however, that the patients in the nine included trials were not exclusively individuals with anxiety disorders or significant anxiety problems. Conclusively, current evidence is very limited and hampers the generalizability of CBT-I to those with anxiety disorders.

### Depression

Since 2008, nine randomised controlled trials (RCTs) have investigated the effects of CBT-I on various measures of sleep and depressive symptomology [11, 23–26, 27••, 28••, 29••, 30••]. When depression has been operationalised as scoring above cut-offs on questionnaires and/or allowing mixed psychiatric diagnoses, medium to large treatment effects on insomnia and sleep have been demonstrated, but the results on depressive outcomes are mixed [11, 23, 25]. Two early studies that included patients with major depressive disorder and investigated CBT-I as an adjunct to pharmacological treatment [24, 26] demonstrated large effects on global sleep measures [Insomnia Severity Index (ISI): Cohen’s *d* = 1.03–1.08] and small to large effects on other sleep measures (sleep diaries and actigraphy: Cohen’s *d* = 0.31–0.84). Statistically significant effects on depression were found in one (Cohen’s *d* = 1.28) [26] but not the other study, although there was a trend towards an effect [24]. The latter study lacked a follow-up and the former had only a 4-week follow-up.

In the recent years, four new RCTs have expanded the field by combining thorough clinical assessments of insomnia and depression, and longer follow-ups, with active psychological control treatments (see Table 1).

**Table 1:** Description of trials on CBT-I in psychiatric disorders

Authors (year)	Sample description	Study design	Interventions: format, frequency and duration	CBT-I components
Ashworth et al. (2015) [27••]	41 participants with major depression and insomnia	RCT (1) Individual CBT-I (2) Self-help CBT-I	Individual interventions, 4 sessions of CBT-I or self-help CBT-I over 8 weeks	Psychoeducation, sleep hygiene, abdominal breathing, stimulus control, sleep restriction, progressive muscle relaxation, cognitive restructuring, imagery relaxation, self-management, relapse prevention and mindfulness
Blom et al. (2015) [28••]	43 participants with major depression and insomnia	RCT (1) CBT-I (2) CBT-D	Internet-based interventions with therapist support, 9 modules over 9 weeks	Psychoeducation, sleep hygiene, education on sleep medication and how to quit, sleep restriction, stimulus control, stress management, managing fatigue, handling negative thoughts about sleep and planning ahead
Clarke et al. (2015) [30••]	41 participants with major depression and insomnia	RCT (1) CBT-I + CBT-D (2) Sleep hygiene + CBT-D	Individual interventions, 3–4 sessions of CBT-I or SH + 4–6 sessions of CBT-D over 12 weeks	Stimulus control, sleep restriction, cognitive techniques to alter unhelpful beliefs regarding insomnia, regularising day/night schedules and savouring
Freeman et al. (2015) [31••]	50 patients with a psychotic disorder with persistent, distressing delusions or hallucinations and insomnia	RCT (1) Standard care plus CBT-I (2) Standard care	Individual interventions, 8 sessions over 12 weeks	Psychoeducation, assessment, goal setting; the following components were delivered optionally, based on the assessment: stimulus control, establishment of appropriate daytime activity and circadian rhythms, sleep hygiene, relaxation training, cognitive techniques and relapse prevention
Gellis et al. [2011] [32]	8 patients with post-traumatic stress disorder and insomnia	Open trial (1) CBT-I	Individual intervention, 5 sessions over 5 weeks	Stimulus control, sleep hygiene, sleep compression and relaxation training
Harvey et al. (2015) [33••]	58 participants with bipolar I and insomnia	RCT (1) CBT-I-BP (2) Psychoeducation	Individual interventions, 8 sessions over 8 weeks	Psychoeducation, case formulation, motivational interviewing, goal setting, stimulus control, sleep restriction, devising wind-down routines in dim light and wake up routines in bright light, cognitive techniques to alter unhelpful beliefs, anxiety, vigilance, worry and rumination, behavioural experiments, and relapse prevention
Myers et al. (2011) [34]	15 patients with a psychotic disorder with persistent persecutory delusions and insomnia	Open trial (1) CBT-I	Individual intervention, 4 weekly or biweekly sessions over 4–8 weeks	Psychoeducation, assessment, goal setting; the following components were delivered optionally (based on the assessment): stimulus control, establishment of appropriate daytime activity and circadian rhythms, sleep hygiene, relaxation training, cognitive techniques and relapse prevention
Norell-Clarke et al. (2015) [29••]	64 participants with major depression or subthreshold depression and insomnia	RCT (1) CBT-I (2) Relaxation training	Group interventions, 4 sessions over 7 weeks	Psychoeducation, sleep hygiene, sleep restriction, stimulus control, cognitive techniques (constructive worry, thought records and cognitive restructuring) and relapse prevention
Talbot et al. (2014) [35••]	45 patients with post-traumatic stress disorder and insomnia	RCT (1) CBT-I (2) Monitor-only waitlist control	Individual interventions, 8 sessions over 8 weeks	Stimulus control, sleep restriction, sleep hygiene, cognitive intervention to address catastrophic beliefs and relapse prevention

*CBT-D* cognitive behavioural therapy for depression, *CBT-I* cognitive behavioural therapy for insomnia, *CBT-I-BP* cognitive behavioural therapy for insomnia–bipolar disorder-specific modification, *RCT* randomised controlled trial, *SH* sleep hygiene

In the first RCT, participants with major depressive disorder and insomnia received either individual CBT-I or self-help CBT-I [27••]. Individual CBT-I was superior to self-help CBT-I and showed large, significant effects on both insomnia severity (ISI: Cohen’s *d* = 0.92) and on depressive symptomology [Beck Depression Inventory-II (BDI-II: Cohen’s *d* = 1.24]. The results had improved further by the time of the 3-month follow-up. Individual CBT-I was also associated with greater improvements on sleep diary measures (Cohen’s *d* = 0.48–1.10). Reliable reductions of at least 7 points on the BDI-II were achieved by 94 % of the participants who had received individual CBT-I compared to 39 % from the self-help group. Also, 78 % of those who had received individual CBT-I had remitted from depression compared to 17 % in the self-help group. Six percent in the self-help group reported a definite worsening of depressive symptoms whereas no adverse outcomes were reported for the individual CBT-I group.

In the second study, participants with major depressive disorder and insomnia received therapist-supported internet CBT-I or therapist-supported internet CBT for depression (CBT-D) [28••]. Both treatments showed medium to large post-treatment within-group effects on insomnia severity (ISI: Cohen’s *d* = 0.54–1.06) and depression severity [Montgomery-Åsberg Depression Rating Scale—self-report version (MADRS-S): Cohen’s *d* = 0.74–0.66] and the effects were maintained or increased by the 6 and 12 month follow-ups. However, CBT-I was superior in reducing insomnia but there were no differences between the treatments regarding depression: neither regarding symptom ratings nor clinical assessments after treatment. There was no significant interaction between group and time for any sleep diary measures. At post-assessment, 40 % of those in the CBT-I group expressed a need for further treatment or insomnia, whereas 80 % of those who had received CBT-D expressed this need. There were no significant differences between CBT-I and CBT-D in the proportions of those needing more treatment for depression (68 vs 81 %). Four participants in each group reported adverse events related to treatment or study requirements.

In the third study (conducted by the current review’s authors with colleagues), participants were randomised to group CBT-I or group relaxation training (RT) [29••]. All participants had insomnia; the majority were diagnosed with major depression whereas the remaining had above 13 on BDI-II and were defined as having subthreshold depression. Post-treatment analyses showed that CBT-I was superior to RT on the ISI (0.79) and some sleep diary measures (sleep onset latency: Cohen’s *d* = 0.42, wake after sleep onset: Cohen’s *d* = 1.06) but there were no significant differences between the groups regarding early morning awakenings, total sleep time or sleep quality. The effects were largely maintained or improved at the follow-up measure. The follow-up CBT-I showed a superior effect in reducing functional impairment [Work and Social Adjustment Scale (WSAS): Cohen’s *d* = 0.44/1.00]. Although the within-group analyses of depressive severity as measured with BDI-II showed that CBT-I had a medium effect (Cohen’s *d* = 0.79) while RT showed a small effect (0.31) at post-treatment, there were no significant differences between treatments. Complementary analyses indicated that those who had received CBT-I and had subthreshold depression had greater improvements on BDI-II (Cohen’s *d* = 0.58) compared to those who received RT but this was not significant (*p* = 0.07). Regarding remission, CBT-I had a greater proportion of remission from both insomnia (65.6 vs 28.1 %) and depression (68.8 vs 43.8 %).

Although CBT-I for comorbid insomnia has been fairly frequently researched on adults, studies on children and adolescents are few. This brings us to the fourth RCT: a pilot study which investigated the effect of individual CBT-I in combination with CBT-D vs sleep hygiene + CBT-D on 12–20 year olds [30••]. Patients had insomnia comorbid with major depression and had follow-ups after treatment and 6 months later. Remarkably, there were no sustained significant differences between the groups on sleep parameters (actigraphy, sleep diary, ISI) or depression questionnaires. As CBT-I was followed directly by CBT-D, any specific effects from CBT-I may have been obscured by the effects from CBT-D. It should also be underscored that the effect sizes were in favour of the CBT-I treatment arm.

### Bipolar Disorder

Only one RCT has investigated the efficacy of CBT-I for those with bipolar disorder (see Table 1). Inter-episode patients with bipolar I were randomised to either an individual treatment package including CBT-I, chronotherapy and social rhythms therapy (CBT-I-BP) or an active control consisting of psychoeducation [33••]. The results showed that CBT-I-BP was associated with a large effect on ISI (Cohen’s *d* = 1.03), a greater proportion of response to treatment (decrease of 8 or more points on ISI: 68.2 vs 28.6 %) as well as remittance from insomnia (ISI and structured clinical interview: 72.7–73.9 % vs 14.3–41.7 %). The results largely remained at the 6-month follow-up. However, there were no differences between the groups for sleep diary outcomes. Functional impairment and quality of life was assessed but there were no group differences. Regarding bipolar disorder, the CBT-I-BP group had fewer days spent in any bipolar episode at follow-up as well as lower mania/hypomania relapse rates as assessed by structured clinical interviews. Regarding relapse to depression, there was no difference between groups, and there were no difference between the groups regarding relapse to any bipolar episode. Symptom severity of hypomania/mania and depression had not changed significantly after treatment but as patients were inter-episode; this was likely due to ceiling effects. No adverse events were reported even though sleep restriction was used. The results indicate that CBT-I-BP has an effect on insomnia and may have a preventive effect of future manic/hypomanic episodes.

Potential adverse effects from core behavioural techniques of CBT-I, sleep restriction and stimulus control, have been investigated in a sample of 15 patients with bipolar I and insomnia [36•]. Although a total of four patients reported elevated hypomanic symptoms after the introduction of the abovementioned behavioural techniques, this was unrelated to their total sleep time. Thus, it is implausible that the symptoms were due to any behaviourally introduced sleep deficit. The effects of CBT-I on patients with bipolar II and patients in active episodes are, however, unknown.

### Post-Traumatic Stress Disorder

Only two studies have explored the efficacy of CBT-I in those with PTSD and comorbid insomnia [32, 35••] (see Table 1). In the first study (open trial without a control group), eight patients with PTSD and comorbid insomnia had individual CBT-I [32]. From pre- to post-treatment, CBT-I resulted in improved sleep, as assessed with sleep diaries (Cohen’s *d* = 0.6–1.4), reduced insomnia severity (Cohen’s *d* = 3.2) and depression severity (Cohen’s *d* = 0.91). Objectively measured sleep (actigraphy) was unchanged across the study period. Also, there were no changes on nightmares (Cohen’s *d* range = up to 0.17) or PTSD severity (Cohen’s *d* range = up to 0.12). In the second trial (RCT), patients were randomised to either individual CBT-I or a monitor-only waitlist control [35••]. Relative to the waitlist control, CBT-I was largely superior in improving sleep from pre- to post-treatment and to 6-month follow-up. The improved sleep in the CBT-I group was evident on the majority of subjective sleep diary parameters (Cohen’s *d* = 0.30–1.48), on one objectively measured outcome (total sleep time), on insomnia severity (Cohen’s *d* = 1.59), overall sleep quality (Cohen’s *d* = 1.43) and sleepiness (Cohen’s *d* = 0.67). While 41 % in the CBT-I group fulfilled the criterion for remission of insomnia, none in the control group met the criterion for insomnia remission. Compared with the control, CBT-I also resulted in reductions in disruptive nocturnal behaviours and overall functioning. Both groups reported reductions in PTSD symptoms and nightmares.

Apart from studies investigating the efficacy of CBT-I in isolation, CBT-I has also been used in combination with imagery rehearsal (IRT), a treatment component designed to target nightmares and not insomnia per se [37]. Investigations of combined treatments (CBT-I + IRT) have demonstrated improvements of nightmares, sleep quality and PTSD symptoms [38–43]. As a whole, combined treatment appears to result in larger improvements than IRT alone [37].

### Psychotic Disorders

Two trials have investigated whether CBT-I is effective for those with a psychotic disorder and comorbid insomnia (see Table 1). In the first study (open trial without a control group), 15 patients were treated with individual CBT-I [34]. CBT-I showed a large, significant effect (Cohen’s *d* = 2.3–2.6) on global insomnia measures (i.e. the Insomnia Severity Index and the Pittsburgh Sleep Quality Index) across the study period (post-treatment and 1-month follow-up), and 67–93 % in the sample demonstrated a reliable or clinically significant change. Following CBT-I, the patients also experienced large, significant reductions in persecutory delusions (i.e. ideas of reference, ideas of persecution, and delusions). Between 47 and 67 % of the patients reported a reliable change and 0–33 % a clinically significant change in persecutory delusions. There were also moderate to large decreases in levels of unusual perceptual anomalies (e.g. distortion of the external world and hallucinations), anxiety and depression. All the improvements were maintained at the 1-month follow-up. No adverse events were reported in the trial.

In the second trial (feasibility RCT), individual standard care (i.e. antipsychotic medication and contact with the local clinical team) plus CBT-I was compared with standard care only [31••]. After the intervention phase, the patients were re-assessed at post-treatment and 3-month follow-up. Relative to standard care only, CBT-I showed a superior effect on insomnia severity and global sleep quality with medium to large effects (Cohen’s *d* = 0.6–1.9) across the study period. At post-treatment, 41 % in the CBT condition and 4 % in the standard care group showed a marked improvement on insomnia severity. Other sleep parameters, as assessed with sleep diaries and actigraphy, showed small to medium effects in both groups (Cohen’s *d* = 0.1–0.6). There were no apparent group differences over time on delusions, hallucinations, paranoia or total symptoms (Cohen’s *d* = 0.1–0.3). Based on the effect sizes, CBT-I appeared more potent in reducing fatigue and increasing quality of life and wellbeing (Cohen’s *d* = 0.3–0.7). Adverse events were reported but were not considered to be related to study treatment.

## Discussion

The purpose of the current paper was to review the effects of CBT-I on psychiatric disorders and to provide suggestions for future research. As we were unable to identify any studies among those with anxiety disorders, our conclusions are limited to those with depression, bipolar disorder, PTSD and psychotic disorders. Across the abovementioned psychiatric disorders, we draw the conclusion that CBT-I, compared to control conditions, clearly reduces insomnia symptomatology, although there were a few exceptions [30••, 31••, 33••] (two of the studies did not show significantly superior improvements on sleep diary parameters). Concerning changes in psychiatric symptoms, the findings were mixed when CBT-I was compared with control conditions; only one study demonstrated significant changes in such outcomes [27••], four investigations did not establish significant improvements [28••, 30••, 31••, 35••] and, finally, two studies provided mixed results within the investigations [29••, 33••]. Four studies used measures to explore changes on daytime symptoms and function [29••, 31••, 33••, 35••], of which only two investigations demonstrated that CBT-I was statistically superior to a control condition [29••, 35••]. A final noteworthy finding was also that a few of the reviewed studies assessed adverse events when delivering CBT-I [27••, 28••, 31••, 33••, 34]; in only one of the five investigations, adverse events were reported to be associated with CBT-I [28••].

Based on neurobiological findings, it has been proposed that if insomnia improves, the comorbid condition should also be improved [44]. Our general conclusion that CBT-I had a limited effect on comorbid psychiatric symptomologies may at first glance seem contradictory to theories regarding insomnia or poor sleep as a maintaining process of psychiatric disorders, but the topic is complex. Although sleep had improved on global measures of insomnia in most of the studies, findings on sleep diaries or actigraphy were mixed. Reporting that one is less troubled by insomnia (as measured by ISI) after treatment may not correspond to decreased objective night-time symptomology. Rather, a decrease in insomnia-related concern may develop naturally over the course of insomnia [45] or from achieving trustworthy information about sleep. Improvements on global sleep measures only may be insufficient to detect objectively improved sleep. One of the reviewed studies specifically examined insomnia as a mechanism of change [27••]. Mediation analyses indicated a reciprocal relationship between improvements in insomnia and depressive severity as changes on either ISI or BDI mediated changes in the other outcome [27••]. Improvements in insomnia had a stronger effect on depression than *vice versa*.

We noticed great variations between studies in CBT-I content. In some of the reviewed studies, one of the most potent tools of CBT-I—sleep restriction—had been omitted [31••, 34]; in other cases, novel treatment components were added to CBT-I, sometimes justified by related research findings or theories regarding specific patient groups (e.g. [33••]). This makes it more difficult to draw conclusions regarding what the results are dependent upon: the specific combination of components or the specific patient group. One could argue that specific patient groups need adjusted CBT-I (e.g. due to sensitivities for sleep deprivation or belonging to different age groups such as adolescents) but that is an empirical question that remains to be answered by RCTs contrasting ‘generic’ CBT-I and adjusted versions. Another variation we noticed between studies regarded cognitive techniques. While some articles included detailed descriptions of specific cognitive techniques and what they were used to target (e.g. [33••]), most studies included vague descriptions. This is problematic as there is a plethora of cognitive techniques that can be used within a CBT-I framework but have varying research support. There are also several rationales behind which techniques to use and for what purpose (e.g. sometimes information is considered a cognitive technique as it is used to counter misunderstandings about sleep, problem-solving techniques can be used to decrease worry, and behavioural experiments can be used to achieve cognitive restructuring). Summarising the findings from studies that have used different CBT-I packages is complicated, especially when there is uncertainty regarding exactly what the treatments included. Clearly, CBT-I is not served in a ‘one size fits all’ format when it comes to comorbid insomnia. Before customising CBT-I for comorbid patient groups, we need to know more about how effective different components are: alone or in combinations. More detailed descriptions of cognitive techniques are also warranted.

## Future Research

This review clearly shows that empirical research on the effects of CBT-I in psychiatric disorders has blossomed in the recent years. There are, however, a number of pertinent limitations in the research area as a whole that we believe need to be addressed in future research. One such limitation is the relatively small number of studies in the area. Two related methodological problems in published studies are also the fairly limited sample sizes and the lack of additional, important outcomes that have not been assessed (e.g. quality of life, overall impairment and cost-effectiveness). Another important question for future research is to examine the effects of CBT-I in those with anxiety disorders and bipolar II disorder, two clinical entities that have so far not been explored. A third limitation with current research is that all published investigations, except one [30••], have recruited and treated participants only from the adult population. Future research also needs to be carried out in children, adolescents and the elderly. A qualitative study that used focus groups with adolescents with comorbid insomnia and depression found indications that specific adjustments may be needed to tailor CBT-I for adolescents regarding both content and format [46•]. A related notion is also the optimal time-point for using CBT-I in those with psychiatric disorders. For example, should CBT be delivered between episodes [33••], resulting in potentially smaller effects on psychiatric symptoms due to floor effects, or when patients are symptomatic, possibly enhancing the risk of dropout, non-compliance and adverse events during CBT-I? Another vital area is to validate and employ active control conditions that are both credible to patients and comparable to CBT-I (e.g. in length as well as in dosage of homework assignments and therapist support). An associated methodological problem is also that it is rare that investigators have assessed the patients’ adherence or compliance to the CBT-I components, acceptability of CBT-I, perceived usefulness of or general satisfaction with CBT-I and credibility of CBT-I. Another important question for future research is to examine the optimal type of delivery for those with psychiatric disorders, e.g. comparing face-to-face CBT-I with group-delivered, internet-based and self-help formats. We also recommend future research to assess and analyse potential moderators (e.g. severity of the psychiatric disorder) and mediators of CBT-I [47].

## Conclusions

Studies investigating the effects of CBT-I on psychiatric populations are vitally important as insomnia is commonly comorbid with psychiatric diagnoses and the comorbidity has been associated with more severe psychopathology. Encouragingly, the CBT-I research field has grown substantially over the recent years and evolved into investigating new comorbid patient groups as well as utilising more advanced study designs. In this review, we conclude that CBT-I was associated with improved insomnia remission and effects on global measures on insomnia, whereas the effects measured by sleep diaries and actigraphy were mixed. Effects on comorbid psychiatric symptomologies were modest: the greatest effects were found in studies without active control groups and there is only one RCT with an active control that demonstrated superiority of CBT-I on psychiatric symptom severity. A tentative clinical conclusion is that CBT-I can be expected to improve comorbid insomnia but the effects on other psychiatric symptoms are unlikely to exceed the effects from other psychological interventions. Further, the generalisability into clinical contexts is hampered by great variations between the psychiatric disorders regarding the number of studies and methodological quality. We look forward to future developments in the areas of the so far neglected psychiatric populations and age groups, as well as methodological designs.

## References

[1] American Psychiatric Association (2013). Diagnostic and statistical manual of mental disorders.

[2] Ford DE, Kamerow DB (1989). Epidemiologic study of sleep disturbances and psychiatric disorders. An opportunity for prevention?. Jama.

[3] Kim B-S, Jeon HJ, Hong JP, Bae JN, Lee J-Y, Lee Y-M (2012). DSM-IV psychiatric comorbidity according to symptoms of insomnia: a nationwide sample of Korean adults. Soc Psychiatry Psychiatr Epidemiol.

[4] Sunderajan P, Gaynes BN, Wisniewski SR, Miyahara S, Fava M, Akingbala F (2010). Insomnia in patients with depression: a STAR*D report. CNS Spectr.

[5] McCall WV, Blocker JN, D’Agostino R, Kimball J, Boggs N, Laseter B (2010). Insomnia severity is an indicator of suicidal ideation during a depression clinical trial. Sleep Med.

[6] Taylor DJ, Lichstein KL, Durrence HH, Reidel BW, Bush AJ (2005). Epidemiology of insomnia, depression and anxiety. Sleep.

[7] Sylvia LG, Dupuy JM, Ostacher MJ, Cowperthwait CM, Hay AC, Sachs GS (2012). Sleep disturbance in euthymic bipolar patients. J Psychopharmacol.

[8] Troxel WM, Kupfer DJ, Reynolds CF, Frank E, Thase M, Miewald J (2012). Insomnia and objectively measured sleep disturbances predict treatment outcome in depressed patients treated with psychotherapy or psychotherapy-pharmacotherapy combinations. J Clin Psychiatry.

[9] Sateia M (2014). International classification of sleep disorders—third edition: highlights and modifications. CHEST J.

[10] Bastien CH, Morin CM, Quellet M-C, Blais FC, Bouchard S (2004). Cognitive-behavioral therapy for insomnia: comparison of individual therapy, group therapy, and telephone consultations. J Consult Clin Psychol.

[11] Lancee J, Van Den Bout J, Van Straten A, Spoormaker VI (2012). Internet-delivered or mailed self-help treatment for insomnia? a randomized waiting-list controlled trial. Behav Res Ther.

[12] Riemann D, Perlis ML (2009). The treatments of chronic insomnia: a review of benzodiazepine receptor agonists and psychological and behavioral therapies. Sleep Med Rev.

[13] Mitchell MD, Gehrman P, Perlis ML, Umscheid CA. Comparative effectiveness of cognitive behavioral therapy for insomnia: A systemantic review. BMC Fam Pract. 2012 13(40). 10.1186/1471-2296-13-40 PMC3481424 22631616

[14] Wu JQ, Appleman ER, Salazar RD, Ong JC (2015). Cognitive behavioral therapy for insomnia comorbid with psychiatric and medical conditions: a meta-analysis. JAMA Intern Med.

[15] Geiger-Brown JM, Rogers VE, Liu W, Ludeman EM, Downton KD, Diaz-Abad M (2015). Cognitive behavioral therapy in persons with comorbid insomnia: a meta-analysis. Sleep Med Rev.

[16] Sánchez-Ortuño MM, Edinger JD (2012). Cognitive-behavioral therapy for the management of insomnia comorbid with mental disorders. Curr Psychiatry Rep.

[17] Suh S. Cognitive behavioral therapy for insomnia: is it effective in treating symptoms of comorbid psychiatric and medical disorders? A review. Sleep Med Res. 2015;6(1):10–5.

[18] Smith MT, Huang MI, Manber R (2005). Cognitive behavior therapy for chronic insomnia occurring within the context of medical and psychiatric disorders. Clin Psychol Rev.

[19] Taylor DJ, Pruiksma KE (2014). Cognitive and behavioural therapy for insomnia (CBT-I) in psychiatric populations: a systematic review. Int Rev Psychiatry.

[20] Dolsen MR, Asarnow LD, Harvey AG (2014). Insomnia as a transdiagnostic process in psychiatric disorders. Curr Psychiatry Rep.

[21] Belleville G, Cousineau H, Levrier K, St-Pierre-Delorme M-È (2011). Meta-analytic review of the impact of cognitive-behavior therapy for insomnia on concomitant anxiety. Clin Psychol Rev.

[22] Ye Y, Zhang Y, Chen J, Liu J, Li X, Liu Y (2015). Internet-based cognitive behavioral therapy for insomnia (ICBT-i) improves comorbid anxiety and depression—a meta-analysis of randomized controlled trials. PLoS One.

[23] Wagley JN, Rybarczyk B, Nay WT, Danish S, Lund HG (2013). Effectiveness of abbreviated CBT for insomnia in psychiatric outpatients: sleep and depression outcomes. J Clin Psychol.

[24] Manber R, Edinger JD, Gress JL, San Pedro-Salcedo MG, Kuo TF, Kalista T (2008). Cognitive behavioral therapy for insomnia enhances depression outcome in patients with comorbid major depressive disorder and insomnia. Sleep.

[25] Vitiello MV, Rybarczyk B, Von Korff M, Stepanski EJ (2009). Cognitive behavioral therapy for insomnia improves sleep and decreases pain in older adults with co-morbid insomnia and osteoarthritis. J Clin Sleep Med.

[26] Watanabe N, Furukawa TA, Shimodera S, Morokuma I, Katsuki F, Fujita H (2011). Brief behavioral therapy for refractory insomnia in residual depression: an assessor-blind, randomized controlled trial. J Clin Psychiatry.

[27] Ashworth DK, Sletten TL, Junge M, Cunnington D, Rajaratnam SM (2014). A randomised controlled trial of cognitive behavioural therapy for insomnia as an adjunct therapy to antidepressants for co-morbid insomnia and depression. Sleep.

[28] Blom K, Jernelöv S, Kraepelien M, Bergdahl MO, Jungmarker K, Ankartjärn L (2015). Internet treatment addressing either insomnia or depression, for patients with both diagnoses—a randomized trial. Sleep.

[29] Norell-Clarke A, Jansson-Fröjmark M, Tillfors M, Holländare F, Engström I (2015). Group cognitive behavioural therapy for insomnia: effects on sleep and depressive symptomatology in a sample with comorbidity. Behav Res Ther.

[30] Clarke G, Mcglinchey EL, Hein K, Gullion CM, Dickerson JF, Leo MC (2015). Cognitive-behavioral treatment of insomnia and depression in adolescents: a pilot randomized trial. Behav Res Ther.

[31] Freeman D, Waite F, Startup H, Myers E, Lister R, Mcinerney J (2015). Efficacy of cognitive behavioural therapy for sleep improvement in patients with persistent delusions and hallucinations (BEST): a prospective, assessor-blind, randomised controlled pilot trial. Lancet Psychiatry.

[32] Gellis LA, Gehrman PR (2011). Cognitive behavioral treatment for insomnia in veterans with long-standing posttraumatic stress disorder: a pilot study. J Aggress Maltreat Trauma.

[33] Harvey AG, Soehner AM, Kaplan KA, Hein K, Lee J, Kanady J (2015). Treating insomnia improves mood state, sleep, and functioning in bipolar disorder: a pilot randomized controlled trial. J Consult Clin Psychol.

[34] Myers E, Startup H, Freeman D (2011). Cognitive behavioural treatment of insomnia in individuals with persistent persecutory delusions: a pilot trial. J Behav Ther Exp Psychiatry.

[35] Talbot LS, Maguen S, Metzler TJ, Schmitz M, McCaslin SE, Richards A (2014). Cognitive behavioral therapy for insomnia in posttraumatic stress disorder: a randomized controlled trial. Sleep.

[36] Kaplan K, Harvey A (2013). Behavioral treatment of insomnia in bipolar disorder. Am J Psychiatry.

[37] Casement MD, Swanson LM (2012). A meta-analysis of imagery rehearsal for post-trauma nightmares: effects on nightmare frequency, sleep quality, and posttraumatic stress. Clin Psychol Rev.

[38] Germain A, Richardson R, Moul DE, Mammen O, Haas G, Forman SD (2012). Placebo-controlled comparison of prazosin and cognitive-behavioral treatments for sleep disturbances in US military veterans. J Psychosom Res.

[39] Germain A, Shear MK, Hall M, Buysse DJ (2007). Effects of a brief behavioral treatment for PTSD-related sleep disturbances: a pilot study. Behav Res Ther.

[40] Harb GC, Cook JM, Gehrman PR, Gamble GM, Ross RJ (2009). Post-traumatic stress disorder nightmares and sleep disturbance in Iraq war veterans: a feasible and promising treatment combination. J Aggress Maltreat Trauma.

[41] Margolies SO, Rybarczyk B, Vrana SR, Leszczyszyn DJ, Lynch J (2013). Efficacy of a cognitive-behavioral treatment for insomnia and nightmares in Afghanistan and Iraq veterans with PTSD. J Clin Psychol.

[42] Nappi CM, Drummond SPA, Hall JMH (2012). Treating nightmares and insomnia in posttraumatic stress disorder: a review of current evidence. Neuropharmacology.

[43] Ulmer CS, Edinger JD, Calhoun PS (2011). A multi-component cognitive-behavioral intervention for sleep disturbance in veterans with PTSD: a pilot study. J Clin Sleep Med.

[44] Harvey AG, Murray G, Chandler R, Soehner A (2011). Sleep disturbance as transdiagnostic: consideration of neurobiological mechanisms. Clin Psychol Rev.

[45] Pillai V, Roth T, Drake CL. Towards quantitative cut-offs for insomnia: how current diagnostic criteria mischaracterize remission. Sleep Med. 2016. 10.1016/j.sleep.2016.01.013 PMC4983260 27288048

[46] Conroy DA, Czopp AM, Dore-Stites D, Dopp RR, Armitage R, Hoban TF (2015). A pilot study on adolescents with depression and insomnia: qualitative findings from focus groups. Behav Sleep Med.

[47] Schwartz DR, Carney CE (2012). Mediators of cognitive-behavioral therapy for insomnia: a review of randomized controlled trials and secondary analysis studies. Clin Psychol Rev.

